# Abnormalities of mucosal serotonin metabolism and 5‐HT_3_ receptor subunit 3C polymorphism in irritable bowel syndrome with diarrhoea predict responsiveness to ondansetron

**DOI:** 10.1111/apt.15420

**Published:** 2019-07-24

**Authors:** David Gunn, Klara Garsed, Ching Lam, Gulzar Singh, Melanie Lingaya, Verena Wahl, Beate Niesler, Amanda Henry, Ian P. Hall, Peter Whorwell, Robin Spiller

**Affiliations:** ^1^ NIHR Nottingham Biomedical Research Centre Nottingham University Hospitals NHS Trust and the University of Nottingham Nottingham UK; ^2^ Nottingham Digestive Diseases Centre, School of Medicine University of Nottingham Nottingham UK; ^3^ Royal Derby Hospital Derby UK; ^4^ Northern General Hospital, Sheffield Teaching Hospitals NHS Foundation Trust Sheffield UK; ^5^ Department of Human Molecular Genetics, Institute of Human Genetics University Hospital Heidelberg and Interdisciplinary Center for Neurosciences (IZN), University of Heidelberg Heidelberg Germany; ^6^ Department of Therapeutics and Molecular Medicine NIHR Nottingham Biomedical Research Centre Nottingham UK; ^7^ Department of Neurogastroenterology University of South Manchester Wythenshawe General Hospital Manchester UK

## Abstract

**Background:**

Irritable bowel syndrome with diarrhoea (IBS‐D) is a common condition, greatly reducing the quality of life with few effective treatment options available.

**Aim:**

To report the beneficial response shown in our trial with the 5‐hydroyxtryptamine (5‐HT) receptor 3 antagonist, ondansetron in IBS‐D

**Methods:**

A randomised, placebo‐controlled, cross‐over trial of 5 weeks of ondansetron versus placebo in 125 patients meeting modified Rome III criteria for IBS‐D as previously described. Patients were compared to 21 healthy controls. 5‐HT and 5‐HIAA were measured in rectal biopsies. Whole gut transit time was assessed using a radio‐opaque marker technique. Whole blood DNA was genotyped for an insertion polymorphism in the promoter region of the serotonin transporter gene *SLC6A4*, as well as single nucleotide polymorphisms (SNPs) of the tryptophan hydroxylase gene *TPH1* and 5‐HT_3_ receptor genes *HTR3A, C* and *E*.

**Results:**

Patients’ biopsies showed significantly higher 5‐HIAA levels (2.1 (1.2‐4.2) pmol/mg protein vs 1.1 (0.4‐1.5) in controls, *P* < .0001). 39 patients used < 4 mg/d (“super‐responders”) while 55 required ≥ 4 mg/d. 5‐HT concentrations in rectal biopsies were significantly lower in super‐responders (21.3 (17.0‐31.8) vs 37.7 (21.4‐61.4), *P* = .0357) and the increase in transit time on ondansetron was significantly greater (15.6 (1.8‐31) hours vs 3.9 (−5.1‐17.9) hours). Stool consistency responders were more likely to carry the CC genotype of the SNP p.N163K rs6766410 of the *HTR3C* gene (33% vs 14%, *P* = .0066).

**Conclusion:**

IBS‐D patients have significant abnormalities in mucosal 5‐HT metabolism. Those with the lowest concentration of 5‐HT in rectal biopsies showed the greatest responsiveness to ondansetron.

## 1 INTRODUCTION

When patients with irritable bowel syndrome with diarrhoea (IBS‐D) are asked to report their most bothersome symptoms, erratic bowel habit and urgency are the most common.^1^ Urgency and the associated occasional faecal incontinence cause significant distress and anxiety, which substantially impair their quality of life. Serotonin (5‐hydroxytryptamine (5‐HT)), a neurotransmitter in the gut has many effects which are relevant to this aspect of IBS, stimulating intestinal secretion and colonic motility.^2^ 5‐HT 3 (5‐HT_3_) receptor antagonists including alosetron, cilansetron and ramosetron which block these effects and slow whole gut transit have been shown in meta‐analysis to be effective treatments for IBS‐D.^3^ Alosetron was withdrawn because of adverse events including severe constipation and ischaemic colitis.^4^ It has now been reintroduced under a FDA‐managed risk evaluation and mitigation strategy but is not widely used. Ramosetron, a potent highly specific 5‐HT_3_ receptor antagonist has been shown to be similarly effective and recently another 5‐HT_3_ receptor antagonist, ondansetron, has also been shown to be effective without causing ischaemic colitis.

Our previous studies which focused on post‐infectious IBS (PI‐IBS), two‐thirds of whom have IBS‐D, had shown an increased number of 5‐HT containing enteroendocrine cells.^5^ We also showed in animal models of PI‐IBS^6^ that colonic mucosal 5‐HT was elevated immediately after infection and 5‐hydroxyindole acetic acid (5‐HIAA)/5‐HT ratios were increased while, serotonin transporter gene (SERT gene *SLC6A4*) mRNA expression was depressed up to 56 days post‐infection, these two changes  suggesting long‐lasting accelerated mucosal 5‐HT turnover. This was associated with enhanced firing of afferent neurons during colonic distension, a feature which could be blocked by ondansetron. This led us to hypothesise that increased 5‐HT availability in the intestinal mucosa could be the driver of symptoms and the explanation of the benefit of 5‐HT_3_ receptor antagonists in IBS‐D.

There are marked individual differences in responsiveness to 5‐HT_3_ receptor antagonists which have been correlated with common polymorphisms in key genes governing the synthesis and reuptake of 5‐HT, as well as the structure of the 5‐HT receptors. Responsiveness to alosetron was shown in one trial to be greater with the homozygous l/l variant of the 5‐HTTLPR (serotonin‐transporter‐linked polymorphic region) of *SLC6A4*.^7^ Furthermore, the gene *TPH1* encoding tryptophan hydroxylase 1, the rate limiting enzyme for serotonin synthesis in enterochromaffin cells of the gut, contains several single nucleotide polymorphisms (SNPs), including rs4537731 and rs211105, which have been reported to predict responsiveness to ramosetron.^8^ Finally, the SNP of *HTR3C* p.N163K rs6766410 predicts chemotherapy‐induced vomiting,^9^ which is known to be driven largely by serotonin. This led us to hypothesize that the sensitivity to ondansetron might in part be dependent on genetic variability due to polymorphisms in these genes.

We now present the biomarkers of mucosal 5‐HT metabolism along with the genetic markers obtained from patients participating in our double‐blind cross‐over trial whose clinical results have been previously reported,^10^ aiming to further characterise IBS‐D patients who respond to ondansetron therapy.

## 2 MATERIALS AND METHODS

### 2.1 Design

The design was as previously described.^10^ In brief, 125 patients with IBS‐D meeting Rome III criteria^11^ were recruited from gastroenterology clinics in Nottingham and Manchester and randomised to receive either 5 weeks of ondansetron, then 5 weeks of placebo or placebo followed by ondansetron, with at least 2 weeks washout between the two treatment periods. Doses were titrated for the first 3 weeks of each treatment period, maintaining a constant dose for the final 2 weeks. Twenty‐one healthy volunteers were recruited as controls for comparison. They completed the same questionnaires as IBS‐D patients, underwent rectal mucosal biopsy and provided normal values for colonic transit studies.

### 2.2 Data collection

The following personal baseline data were collected: age, sex, Hospital Anxiety and Depression Scale (HADS)^12^ score, Patient Health Questionnaire 12 Somatic Symptom (PHQ‐12)^13^ score and Perceived Stress Scale^14^ score. IBS Quality of Life Questionnaire^15^ and IBS Severity Scoring System^16^ scores were collected at baseline and at the end of each treatment period. A previously described daily stool diary^17^ was used to collect stool form information (using the Bristol Stool Form Score (BSFS),^18^ abdominal pain perception, urgency to defecate and abdominal bloating (all three scored on a 0‐3 scale). Frequency of defecation and number of days with bloating, pain or urgency were recorded. Whole gut transit time was measured after 5 weeks of each treatment using the Metcalf's radio‐opaque marker technique.^19^

### 2.3 Biopsy analysis

All 70 patients recruited from Nottingham were invited to undergo unprepared flexible sigmoidoscopy to obtain high rectal/sigmoid biopsies and 57 consented to this additional procedure. Two of the samples were immediately frozen in liquid nitrogen for analysis of serotonin and 5‐HIAA content by high‐performance liquid chromatography (HPLC), two in RNALater for gene expression studies, one for incubation at 25°C in 95% O_2_ for assay of 5‐HT release from a 0‐30 minute period as previously described^20^ (Data S1). Finally, one biopsy was embedded in paraffin for routine histology to exclude microscopic colitis. Real‐time polymerase chain reaction (PCR) was used to assess the relative expression of *TPH1* with respect to the housekeeping gene hypoxanthine phosphoribosyltransferase 1 (*HPRT1*). RNA was extracted and analysed according to the previously reported method.^21^

### 2.4 Plasma 5‐HIAA

We assessed fasting plasma 5‐HIAA using HPLC as previously described.^22^

### 2.5 Genotyping

Blood samples were taken at visit 1. DNA was extracted from 200 µl of citrated blood using the QIAamp DNA Blood Mini Kit (Qiagen Cat. No. 51106) and subsequently genotyped for 62 patients who provided samples for the following polymorphisms: 5‐HTTLPR (serotonin‐transporter‐linked polymorphic region) residing within the promoter region of the serotonin transporter gene *SLC6A4* as described previously.^23^ Genotyping of the SNPs within the tryptophan hydroxylase gene *TPH1* rs211105 (noncoding SNP), rs4537731 (noncoding SNP), and in 5‐HT_3_ receptor genes including *HTR3A* c.‐42C > T (rs1062613), *HTR3B* p.Y129S (rs1176744), *HTR3C* p.N163K (rs6766410) and *HTR3E* c.*76G > A (rs56109847) was carried out by the KASPar® assay (KBiosciences, Ltd, Hoddesdon, United Kingdom) using KASP by design primer mixes as recommended by the manufacturer. Thermal cycling was performed in Mastercycler vapo.protect thermal cyclers (Eppendorf). An initial 15‐minute incubation at 95°C was followed by 20 cycles consisting of 10 s at 94°C, 5 s at 57°C and 10 s at 72°C, followed by 23 cycles consisting of 10 s at 94°C, 5 s at 57°C and 10 s at 72°C. After thermal cycling, results were analysed using the fluorescence plate reader of the 7500 Fast Real‐Time PCR System (Applied Biosystems, Foster City, California). About 10% of the samples were repeated to ensure genotyping accuracy.

### 2.6 Responder definition

Our original study was a pilot study, so we used stool form rather than pain as our primary endpoint since it was known to have had a much lower placebo response rate. Therefore, in this paper, we used the US Food and Drug Administration (FDA) definition of a “stool consistency responder” as a “patient who experiences a 50 percent or greater reduction in the number of days per week with at least one stool that has a consistency of Type 6 or 7 compared with baseline”.^24^ Our pain assessments on a 0‐3 scale did not allow us to calculate a pain responder rate according to FDA guidelines which were published after our study was initiated.

### 2.7 Data analysis

Analysis was performed using Graphpad Prism version 7.0c (GraphPad Software, La Jolla California, USA). Efficacy parameters were calculated for each patient as the difference in the endpoints measured in the last 2 weeks of the ondansetron and placebo periods.

Baseline and rectal biopsy data were analysed for differences between patients and healthy volunteers using unpaired t‐tests and Mann‐Whitney tests on parametric and nonparametric data, respectively. Subsequent analysis based on their stool form responder status and mean ondansetron dose was done as below.

Genotype data was correlated with stool form responder status, baseline clinical features, biopsy results, final ondansetron dose, whole gut transit time and *TPH1* mRNA expression. Chi‐squared tests were performed for stool form responder status, and one‐way ANOVA and Kruskal‐Wallis tests were performed on parametric and nonparametric data respectively.

## 3 RESULTS

### 3.1 Participants

Healthy volunteers were age and sex matched to patients. Mean patient and control ages were 41 and 43 years and percentage female were 71% and 76% respectively. The patients taking part in the trial were, as expected, more anxious, more stressed and showed greater somatic symptoms with significantly higher HAD, perceived stress scale and PHQ‐12 scores compared to controls (Table 1). There were no differences between patients consented for biopsy and those who did not. As required for entry into the trial^10^ IBS‐D patients reported significantly higher bowel frequency.

**Table 1:** Demographics of study participants showing details of patients’ and healthy volunteers’ mental health and bowel function. There were no significant differences between those undergoing biopsy and those choosing not to have this extra test

Variable	Patients without biopsy (n = 68)	Patients with biopsy (n = 57)	Heathy volunteers (n = 21)	*P* value
Age	40 (12)	42 (12)	43 (18)	.5921
Sex (women), n (%)	53 (78)	36 (63)	16 (76)	.1693
Patient Health Questionnaire 12	8 (3.5)	7.3 (3.7)	2.2 (1.8)	<.0001
Anxiety	9.8 (4.3)	9.5 (4.8)	5.2 (2.7)	.0001
Depression, median (IQR)	5.5 (3‐10)	4 (2‐8.8)	1 (1‐2)	<.0001
Hospital Anxiety and Depression Scale	16.3 (7.4)	14.8 (8.2)	6.8 (3.3)	<.0001
Perceived Stress Scale	19 (7.5)	17.5 (7.9)	11.2 (6.0)	.0003
Bowel frequency, median (IQR)	2.6 (1.9‐4)	2.8 (2‐4)	1.1 (1‐1.4)	<.0001
Stool form	5.4 (0.8)	5.4 (0.6)	3.5 (0.7)	<.0001

Note

Data are mean (SD), unless stated. *P* values, were significant, demonstrate differences between the patient population and healthy volunteers with no difference between the two patient groups. *P* values are obtained from one‐way ANOVA and Kruskal‐Wallis tests for parametric and nonparametric data respectively.

### 3.2 Rectal biopsies

**Figure 1:**
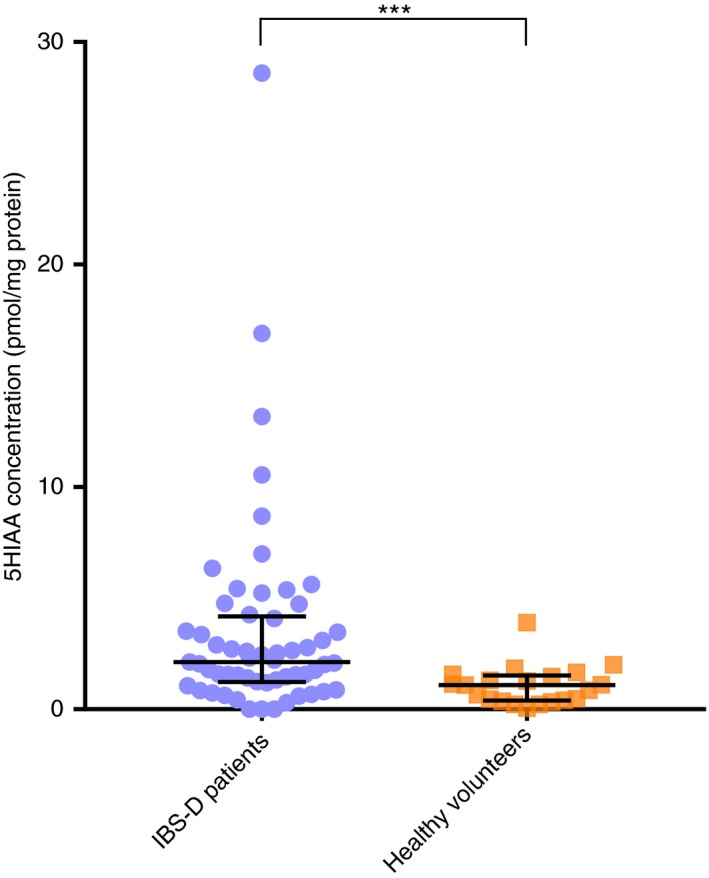
Colonic biopsy 5‐hydroxyindole acetic acid (5‐HIAA) concentrations (median, interquartile range) in pmol/mg protein in IBS‐D patients (n = 57) and healthy volunteers (n = 21); ***P = 0.0001

Rectal biopsies were obtained from 57 of the 125 patients completing the trial and 21 controls. Biopsy 5‐HIAA levels were significantly higher in IBS‐D patients (Figure 1) as were the 5‐HIAA /5‐HT ratios (Table 1). There were, however, no significant differences between patients and controls for biopsy 5‐HT nor 5‐HT release in the first 30 minutes (see Table 2).

**Table 2:** Colonic biopsy 5‐HIAA, 5‐HT, 5‐HIAA/5‐HT, and 5‐HT release during 30 min incubation for IBS‐D patients and healthy volunteers

Variable	Patients (n = 57)	Healthy volunteers (n = 21)	*P* value
biopsy 5‐HIAA (pmol/mg protein)	2.1 (1.2‐4.2)	1.1 (0.4‐1.5)	.0001
biopsy 5‐HIAA/5‐HT	0.06 (0.03‐0.16)	0.02 (0.02‐0.05)	.0038
biopsy 5‐HT (pmol/mg protein)	31.2 (20.1‐46.0)	39.1 (16.5‐46.7)	.9288
biopsy 5‐HT release in 30mins (pmol/mg wet weight)	17.2 (13.5‐23.1)	23.8 (13.8‐44.2)	.078
Biopsy 5‐HT release in 30mins/ Biopsy 5‐HT content	0.8 (0.3‐1.1)	0.8 (0.2‐2.2)	.851

Note

Data are median (IQR). *P* values obtained from Mann‐Whitney tests.

### 3.3 Plasma 5‐HIAA

Despite higher biopsy levels, median plasma 5‐HIAA (nmol/l) was significantly lower in patients vs controls (16.2 vs 20.8, *P* < .0001).

### 3.4 Stool form responders

107/125 IBS‐D patients completed daily stool diaries to allow assessment of response, of which 82 patients met FDA criteria for stool form responders. As shown in Table 3, they had significantly lower baseline average abdominal pain scores and pain occurred on fewer days per week. They also reported lower average urgency scores and urgency on fewer days per week. Responders tended to be younger, with lower HAD scores and to have slower colonic transit; however, these differences failed to reach conventional statistical significance. There were no significant differences in either baseline stool form or frequency (Data S1).

**Table 3:** Demographics, psychological assessments, stool diary results and markers of serotonin turnover in patients subdivided into those who met FDA “stool form responder” criteria and those who did not

Variable	Stool form responder (n = 82)	Stool form nonresponder (n = 25)	*P* value
Age	40 (12)	45 (11)	.0539
Sex (female), n (%)	61 (74)	16 (64)	.446
Hospital Anxiety and Depression Scale	14.7 (7.0)	17.8 (8.3)	.0774
Patient Health Questionnaire 12	7.4 (3.4)	8.2 (3.9)	.3189
Placebo whole gut transit (hours), median (IQR)	18 (9‐31)	10 (6.3‐24.8)	.0625
Baseline days with abdominal pain, median (IQR)	5 (3‐7)	7 (5‐7)	.0175
Baseline average abdominal pain score	1.2 (0.7)	1.8 (0.8)	.0023
Baseline days with urgency, median (IQR)	6 (4.8‐7)	7 (6.5‐7)	.0014
Baseline average urgency score, median (IQR)	1.4 (1‐2)	2.4 (1.6‐2.6)	<.0001
Baseline days with bloating, median (IQR)	6 (3‐7)	6 (3.5‐7)	.498
Baseline average bloating score	1.3 (0.8)	1.4 (0.9)	.4438
Baseline average stool form	5.3 (0.7)	5.5 (0.8)	.2498
Baseline average stool frequency, median (IQR)	2.6 (1.9‐3.9)	2.9 (1.9‐3.9)	.7266
Biopsy 5‐HT (pmol/mg protein), median (IQR)^a^	28.6 (18.3‐42)	44.3 (25.5‐65.7)	.0642
Biopsy 5‐HIAA (pmol/mg protein), median (IQR)^a^	2.1 (1‐4.1)	1.6 (1‐4)	.6567
Biopsy 5‐HIAA /5‐HT, median (IQR)^a^	0.06 (0.03‐0.20)	0.03 (0.02‐0.08)	.2731
Plasma 5‐HIAA (nmol/l), median (IQR)^b^	16.5 (13.1‐19.8)	16.3 (12.7‐18)	.7022

Note

Data are mean (SD) unless stated. *P* values obtained from unpaired *t* tests and Mann‐Whitney tests for parametric and nonparametric data respectively.

Abbreviations: 5‐HT, serotonin; 5‐HIAA, 5‐Hydroxyindoleacetic acid.

a Numbers in each group: n = 40 stool form responder; n = 9 stool form nonresponder.

b Numbers in each group: n = 53 stool form responder; n = 10 stool form nonresponder.

### 3.5 Features of patients showing increased responsiveness to ondansetron (super‐responders)

About 39 IBS‐D patients adjusted their daily dose to < 4 mg ondansetron per day, compared to 55 requiring a dose of ≥ 4 mg ondansetron. Patients on < 4 mg/d (super‐responders) were more commonly female (92% vs 58%, *P* = .003) and had slightly firmer baseline stools (5.1 vs 5.4, *P* = .0159). Other baseline clinical features including pain, urgency and bloating as assessed from days with each symptom and average severity scores; HADS, anxiety, depression and PHQ‐12 scores were not significantly different (see supplementary data).

**Figure 2:**
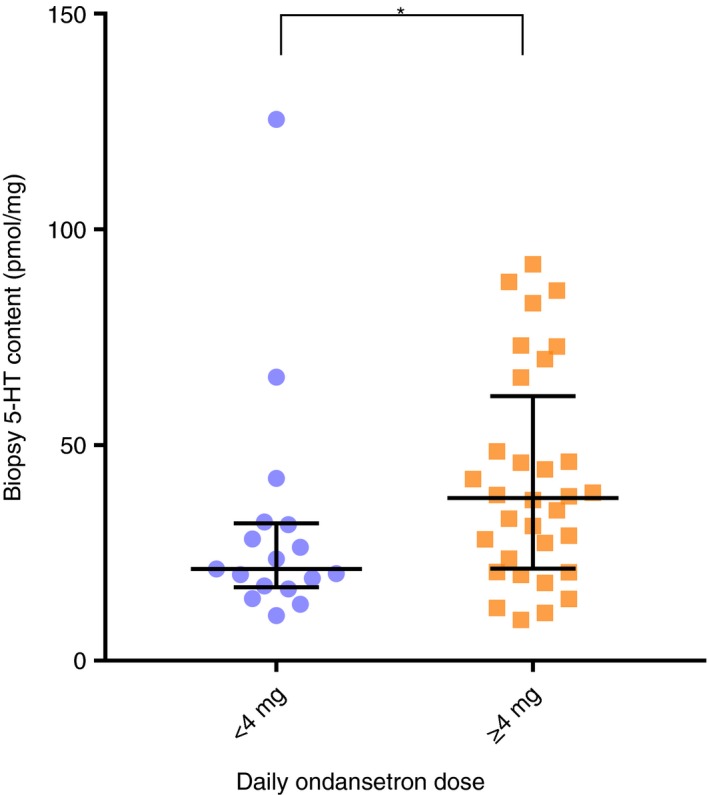
Biopsy serotonin (5‐HT) content (median, interquartile range) in pmol/mg protein subdivided by daily dose showing a significantly lower biopsy 5‐HT content in patients taking < 4 mg (n = 17) compared with those taking ≥ 4 mg ondansetron daily (n = 32), *P* = 0.0357

**Figure 3:**
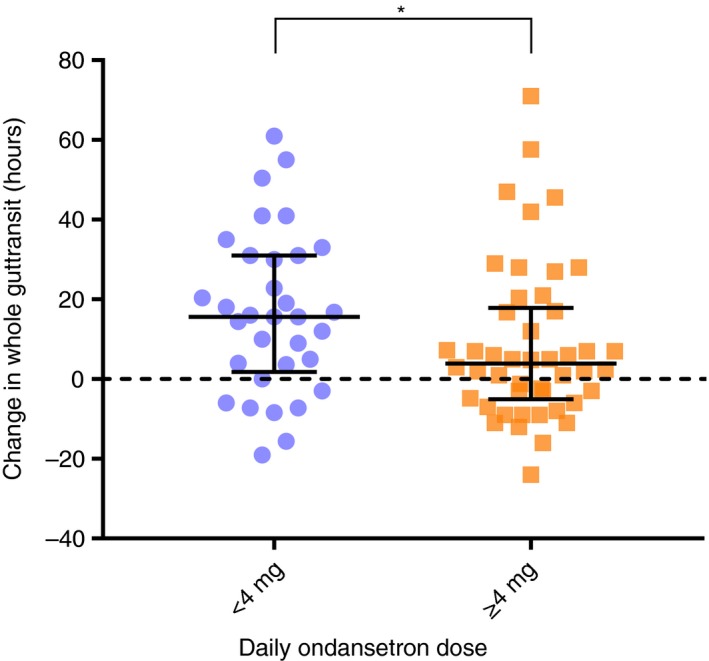
Difference in whole gut transit (median, interquartile range in hours) while taking ondansetron compared to placebo treatment, subdivided into those taking < 4 mg (n = 33) and those taking ≥ 4 mg (n = 46) ondansetron daily. Despite taking a lower dose those taking < 4 mg showed a significantly greater increase in transit time, *P* = 0.0398

There were, however, significant differences in 5‐HT concentration in rectal biopsies which were significantly lower in super‐responders (21.3 (17.0‐31.8) vs 37.7, (21.4‐61.4), *P* = .0357) (Figure 2). There were no differences between the groups in biopsy 5‐HIAA, 5‐HIAA /5‐HT, plasma 5‐HIAA and biopsy *TPH1* mRNA expression (Table 4). However super‐responders, despite the lower dose, showed a fourfold greater increase in whole gut transit time when on ondansetron compared to placebo (15.6 vs 3.9, *P* = .0398) (Figure 3).

**Table 4:** Colonic transit and markers of colonic mucosal serotonin turnover in IBS‐D patients, subdivided into those requiring < 4 mg (super‐responders) and those requiring ≥ 4 mg/d of ondansetron

Variable	<4 mg ondansetron (n = 17)	≥4 mg ondansetron (n = 32)	*P* value
Biopsy 5‐HT (pmol/mg protein)	21.3 (17.0‐31.8)	37.7 (21.4‐61.4)	.0357
Biopsy 5‐HT release in 30 min (pmol/mg wet weight)	23.8 (10.9‐26.4)	17.1 (14‐21.8)	.23
Biopsy 5‐HT release in 30 min/ Biopsy 5‐HT content	1.1 (0.6‐2.1)	0.4 (0.3‐0.8)	.0818
Plasma 5‐HIAA (nmol/l)^a^	17 (14.2‐20.0)	16.0 (12.3‐18.1)	.1733
Biopsy *TPH1* mRNA relative expression^b^	0.61 (0.54‐0.80)	0.61 (0.42‐0.95)	.6554
Placebo whole gut transit time (hours)^c^	18 (7‐30)	14 (7‐29)	.7545
Difference in whole gut transit time on ondansetron versus placebo (h)^d^	15.6 (1.8‐31)	3.9 (−5.1‐17.9)	.0398

Note

Data are median (IQR). *P* values obtained from Mann‐Whitney tests.

Abbreviations: 5‐HT, serotonin; 5‐HIAA, 5‐Hydroxyindoleacetic acid; TPH1, Tryptophan hydroxylase 1.

a Numbers in each group: n = 22 <4 mg ondansetron; n = 41 ≥4 mg ondansetron.

b Numbers in each group: n = 17 <4 mg ondansetron; n = 37 ≥4 mg ondansetron.

c Numbers in each group: n = 35 <4 mg ondansetron; n = 51 ≥4 mg ondansetron.

d Numbers in each group: n = 33 <4 mg ondansetron; n = 46 ≥4 mg ondansetron.

### 3.6 Effect of genotype on treatment response

Stool form responders were more likely to carry the homozygous CC genotype p.N163K rs6766410 of the *HTR3C* gene which was found in 33% of responders compared to 14% of nonresponders, (*P* = .0066, Table 5). The polymorphism in *HTR3E* c.*76G > A rs561098476 showed a trend towards association with more responders carrying the GG genotype compared to nonresponders, although this was nonsignificant (*P* = .09, see supplementary data). Patients carrying the genotype TT of the *HTR3A* SNP rs1062613 reported significant improvement of both days with bloating (1.7 (2.6) vs 5.0 (2.5), *P* = .035) and average bloating score (0.38 (0.7) vs 1.4 (1.0), *P* = .018). The significances were only found comparing the genotypes TT vs CT, and not with CT vs CC or TT vs CC, probably due to the small number of TT individuals (see supplementary data). No other significant associations with any of the genotypes tested were found for the secondary endpoints such as 5‐HT or 5‐HIAA levels, or 5‐HIAA/5‐HT ratio (Table S1). Surprisingly no significant effect was seen for the two *TPH1* SNPs on *TPH1* mRNA levels (Data S1).

**Table 5:** Effect of the polymorphism *HTR3C* p.N163K rs6766410 on IBS‐D patients’ FDA stool responder status, expressed as a percentage of responder status patient population; and clinical features while on ondansetron

Variable	AA genotype (n = 15)	CA genotype (n = 54)	CC genotype (n = 28)	*P* value
Stool responder (n = 22), %	14	53	33	.0066
Stool nonresponder (n = 73), %	18	68	14	
Average stool form, mean (SD)	3.6 (1.5)	4.1 (1.3)	3.9 (1)	.5143
Average stool frequency	2 (1.1‐3.1)	1.8 (1.4‐2.9)	1.6 (1.2‐2.4)	.5892
Days with abdominal pain	4 (1.4‐6.5)	6 (2.5‐7)	5 (1.5‐6.5)	.0923
Average abdominal pain score	0.6 (0.2‐1.4)	1.4 (0.6‐2.1)	0.9 (0.3‐1.3)	.0241
Days with urgency	3.8 (2.3‐6.6)	5 (2.4‐6.5)	3 (1‐6.3)	.2863
Average urgency score, mean (SD)	1.2 (1)	1.2 (0.8)	0.8 (0.7)	.1276
Days with bloating	5 (2‐6.5)	5.8 (1.9‐7)	3 (0.5‐6.3)	.207
Average bloating score	0.8 (0.1‐1.4)	1.4 (0.3‐2.1)	0.6 (0.1‐1.5)	.1636

Note

Data are median (IQR) unless stated. *P* values for clinical features while on ondansetron are obtained from one‐way ANOVA and Kruskal‐Wallis tests for parametric and nonparametric data respectively. For FDA stool responder and polymorphisms the *P* value is obtained from a Chi‐squared test.

### 3.7 TPH1 mRNA expression

Seventy‐eight patient rectal biopsies were analysed for *TPH1* mRNA expression levels. There were no significant correlations with baseline clinical features, biopsy 5‐HT or biopsy 5‐HIAA concentrations (Data S1).

## 4 DISCUSSION

Several previous studies examining different aspects of serotonin metabolism have indicated that this is disturbed in IBS‐D patients with greater post‐prandial 5‐HT in platelet‐poor plasma^22^, ^25^ and reduced SERT mRNA levels in both duodenal^20^ and colonic biopsies in some^26^ but not all^27^ studies. The current study found significantly increased mucosal concentrations of the 5‐HT metabolite 5‐HIAA together with an increased 5‐HIAA/ 5‐HT ratio suggesting greater turnover of mucosal 5‐HT in IBS‐D patients. Previous evidence excess 5‐HT stimulates secretion and motility, this provides a rationale for the use of both *TPH1* inhibitors^28^ and 5‐HT_3_ receptor antagonists in IBS‐D. The effectiveness of 5‐HT_3_ receptor antagonists has been confirmed in meta‐analyses^3^ which show particular benefit for urgency and loose stools.^29^ However, the number of patients needed to treat to get one responder more than with placebo is 7.7, indicating that there are subgroups of individual patients who respond better, as is true of all IBS treatments. Our initial trial, which uniquely allowed patients to select their optimum dose, showed striking differences in the doses chosen, with over half using 4 mg or less per day while some used the maximum allowed of 24 mg/d. These differences suggest that there existed a subgroup with greater sensitivity to ondansetron which we have termed “super‐responders”. We, therefore, looked to see what biomarkers predicted “super‐responder status”.

We found that rectal biopsies of the super‐responders had a significantly lower 5‐HT concentration than those requiring larger doses. When the 5‐HT released from the biopsies over 30 minutes was expressed as a percentage of the biopsy 5‐HT concentration, there was a trend for this to be elevated but this was not statistically significant owing to wide variability in this measure. Comparing Tables 2 and 4 it can be seen that healthy volunteers’ mucosal 5‐HT values were similar to the group requiring ≥ 4 mg. This suggests that it is the super‐responders who have abnormally low mucosal 5‐HT though the other 5‐HT parameters were similar to both healthy controls and the other patients.

The super‐responders showed a four‐fold greater change in transit even although they were taking a dose which was a quarter of that observed in those requiring larger doses, again objectively supporting the idea that super‐responders are very much more sensitive to the drug. Having less serotonin in the biopsy could be either because of reduced synthesis or accelerated turnover. The tendency for increased 5‐HT release when expressed as a percentage of biopsy serotonin content, though just failing to reach conventional statistical significance, would tend to support this interpretation and might explain why they were so much more sensitive to ondansetron if excess 5‐HT was driving their symptoms. Future studies might use in vivo assessment of 5‐HT release as has recently been done in animals to address this issue.^30^

The striking slowing of transit seen in super‐responders has important implications. We have previously reported from this same patient group that the faecal protease levels correlated negatively with transit time and positively with average urgency in IBS‐D patients.^31^ As others have confirmed, urgency is strongly related to fast transit in IBS‐D^32^ but just exactly why slowing transit helps is uncertain. We hypothesise that, by allowing more time for the colonic microbiota to deconjugate bile acids and degrade endogenous proteases,^31^ the slowing of transit may prevent the sensitisation of the rectum that these endogenous irritants can cause. However, further intervention trials using other nonserotonergic agents to slow transit, such as loperamide, will be needed to decide what is unique about the 5‐HT_3_ receptor antagonist's action.

Our finding that stool form responders were more likely to carry the CC genotype p.N163K rs6766410 of *HTR3C* fits with the data of Fasching *et al* 9 which suggested CC was more sensitive to chemotherapy‐induced vomiting, known to be driven largely by serotonin. This substitution has a predicted possible functional effect on the receptor (polyphen2^33^ score 0.798). If those with CC genotype are more sensitive to the effects of 5‐HT this might help explain the benefit of a drug which blocks its action.

Like many mechanistic studies which make substantial demands on patients we were probably underpowered for many of our secondary endpoints. Since our study was carried out on patients referred to secondary care they can only be generalised to this population which may differ from that seen in primary care. We only requested biopsies for patients recruited in Nottingham where the laboratory facilities and staff needed were available and we allowed patients to choose whether to have the extra biopsies. We felt that this was important to facilitate recruitment to the main trial and as Table 1 shows, we did not find any differences between patients who opted to have biopsies compared to those who did not, so we do not think this introduced any bias.

Although we chose to study variants in the *HTR3C* gene with previous evidence for potential influence on response to 5‐HT, these findings need to be interpreted with caution as none of the genetic analyses were corrected for multiple testing and being post hoc need to be replicated before they can be accepted. We were unable to link known SNPs in the genes encoding the SERT gene *SLC6A4* or *TPH1* with a clinically significant pattern of symptom features nor responsiveness to ondansetron, as has been suggested by others for the related drug ramosetron,^34^ but our numbers were probably too low for such an analysis. Also relevant is the fact that we did not find a correlation between *TPH1* SNPs examined and mRNA levels for *TPH1*. These data are compatible with data in Gtex^35^ showing that rs21105 has no significant eQTL signatures. rs4537731 has significant eQTL signatures in skin and thyroid but not in nerve or gut tissue.

Our pilot study asked subjects for pain scores on a 0‐3 scale, which is insufficient to define a decrease in pain by at least 30% as recommended by the FDA. Consequently, we defined response by the change in stool consistency which is suboptimal in an IBS study. Future studies should take this into account by using an 11‐point pain scale allowing an assessment using both pain and stool consistency response according to FDA guidance.

While psychological factors are thought to be important in IBS we found no differences in anxiety nor somatisation between super‐responders and non‐super‐responders, in keeping with ondansetron's known lack of central effects.

Overall, our study is important for highlighting the heterogeneity of IBS‐D and indicates that personalised medicine approach is both necessary and possible if we know the mode of action and can easily assess the key factors determining response to particular drugs. Rectal biopsy 5‐HT might be such a parameter for 5‐HT receptor 3 antagonists, however, rectal biopsy assessment of 5‐HT turnover is not currently a feasible clinical test. The immediate value of our findings is that it encourages us to further subdivide IBS‐D. Some plainly have serotonin excess, but others appear to have quite different mechanisms such as excessive secretions or impaired absorption which we should explore further. Examining the response to 4 mg daily of ondansetron could be a simple test to identify at least one subgroup which is not currently readily identifiable. Future studies of other nonserotonergic agents might use this, as we currently do with a trial of colestyramine, to exclude patients with a known mechanism of diarrhoea and thus improve response rates to the newer agents under trial.

## DISCLAIMER

This is a summary of independent research funded by the National Institute for Health Research Biomedical Research Unit. The views expressed are those of the author(s) and not necessarily those of the NHS, the NIHR or the Department of Health.

## AUTHORSHIP

*Guarantor of the article*: RS.

*Author contributions*: RS, PW and KG, design and conception of trial; KG, CL, PW and RS, Subject recruitment and trial oversight; GS, ML, AH and VW, analysis of samples; DG, RS, BN, IH, KG, data interpretation and writing of manuscript; RS, Manuscript guarantor; All authors, final review prior to submission.
