# Supplementary material for: Abnormalities of mucosal serotonin metabolism and 5‐HT3 receptor subunit 3C polymorphism in irritable bowel syndrome with diarrhoea predict responsiveness to ondansetron
Source: Aliment Pharmacol Ther. 2019 Jul 24;50(5):538–46. doi: 10.1111/apt.15420 (PMC6772086; doi:10.1111/apt.15420)
Supplement: Supplementary file 1 [file APT-50-538-s001.docx]

# Supplementary data

## Supplementary Table 1 – Effect of IBS-D patients’ genotypes on FDA stool responder status (expressed as a percentage of responder status patient population) and clinical features while on ondansetron

*HTR3A* c.-42C>T rs1062613

| **Variable** | **TT genotype (n=7)** | **CT genotype (n=35)** | **CC genotype (n=59)** | ***P* value** |
| --- | --- | --- | --- | --- |
| responder (n=75), % | 8 | 35 | 57 | 0.4806 |
| non-responder (n=24), % | 4 | 38 | 58 |  |
| average stool form, mean (SD) | 4.2 (1.2) | 4 (1.3) | 3.9 (1.3) | 0.6889 |
| average stool frequency | 1.5 (1.2-1.9) | 1.9 (1.4-3.2) | 1.7 (1.1-2.4) | 0.3383 |
| days with abdominal pain | 3 (0-7) | 5.5 (4-6.5) | 4.5 (1.5-7) | 0.4328 |
| average abdominal pain score | 0.5 (0-1.9) | 1.1 (0.9-2) | 0.9 (0.2-1.9) | 0.4005 |
| days with urgency | 2 (0.5-5.5) | 4.5 (1.5-6.5) | 4 (1.5-6.5) | 0.4736 |
| average urgency score | 0.4 (0.1-1.6) | 0.9 (0.4-1.5) | 0.8 (0.3-1.8) | 0.708 |
| days with bloating | 0.5 (0-3.5) | 6 (3-7) | 4 (0.5-6.5) | 0.0162 |
| average bloating score | 0.1 (0-0.5) | 1.4 (0.6-2.2) | 0.8 (0.1-1.8) | 0.0094 |

*HTR3B* p.Y129S rs1176744

| **Variable** | **AA genotype (n=11)** | **CA genotype (n=45)** | **CC genotype (n=88)** | ***P* value** |
| --- | --- | --- | --- | --- |
| responder (n=23), % | 11 | 45 | 45 | 0.888 |
| non-responder (n=74), % | 13 | 43 | 43 |  |
| average stool form, mean (SD) | 4.4 (1.2) | 3.9 (1.4) | 3.9 (1.3) | 0.355 |
| average stool frequency | 1.9 (1.7-2.5) | 1.8 (1.2-3.3) | 1.7 (1.3-2.5) | 0.3951 |
| days with abdominal pain | 6 (4-6.5) | 6 (2.5-7) | 5.5 (2-7) | 0.6065 |
| average abdominal pain score | 1.3 (0.9-1.6) | 1 (0.5-2) | 1 (0.3-1.9) | 0.576 |
| days with urgency | 4.5 (3-7) | 4 (1.5-6.5) | 4 (1.5-6.5) | 0.5695 |
| average urgency score | 0.8 (0.7-1.6) | 0.8 (0.3-1.8) | 0.8 (0.3-1.8) | 0.6173 |
| days with bloating | 5 (2.5-6) | 6 (1.5-7) | 5 (0.6-7) | 0.555 |
| average bloating score | 1 (0.6-1.4) | 1.3 (0.3-2) | 0.9 (0.1-1.9) | 0.5358 |

*HTR3C* p.N163K rs6766410

| **Variable** | **AA genotype (n=15)** | **CA genotype (n=54)** | **CC genotype (n=28)** | ***P* value** |
| --- | --- | --- | --- | --- |
| responder (n=22), % | 14 | 53 | 33 | 0.0066 |
| non-responder (n=73), % | 18 | 68 | 14 |  |
| average stool form, mean (SD) | 3.6 (1.5) | 4.1 (1.3) | 3.9 (1) | 0.5143 |
| average stool frequency | 2 (1.1-3.1) | 1.8 (1.4-2.9) | 1.6 (1.2-2.4) | 0.5892 |
| days with abdominal pain | 4 (1.4-6.5) | 6 (2.5-7) | 5 (1.5-6.5) | 0.0923 |
| average abdominal pain score | 0.6 (0.2-1.4) | 1.4 (0.6-2.1) | 0.9 (0.3-1.3) | 0.0241 |
| days with urgency | 3.8 (2.3-6.6) | 5 (2.4-6.5) | 3 (1-6.3) | 0.2863 |
| average urgency score, mean (SD) | 1.2 (1) | 1.2 (0.8) | 0.8 (0.7) | 0.1276 |
| days with bloating | 5 (2-6.5) | 5.8 (1.9-7) | 3 (0.5-6.3) | 0.207 |
| average bloating score | 0.8 (0.1-1.4) | 1.4 (0.3-2.1) | 0.6 (0.1-1.5) | 0.1636 |

*HTR3E* c.*76G>A rs561098476

| **Variable** | **AA genotype (n=2)** | **GA genotype (n=16)** | **GG genotype (n=80)** | ***P* value** |
| --- | --- | --- | --- | --- |
| responder (n=72), % | 1 | 14 | 85 | 0.0941 |
| non-responder (n=24), % | 0 | 25 | 75 |  |
| average stool form, mean (SD) | 5.2 (0.1) | 4 (1.5) | 4 (1.2) | 0.4037 |
| average stool frequency | 1.5 (1.1-1.9) | 1.9 (1.4-3.3) | 17 (1.3-2.6) | 0.6747 |
| days with abdominal pain | 4.3 (1.5-7) | 6 (1.4-7) | 5.3 (2-6.5) | 0.9024 |
| average abdominal pain score | 1.3 (0.4-2.3) | 1.2 (0.3-2.1) | 1 (0.3-1.8) | 0.761 |
| days with urgency | 4.3 (1.5-7) | 3.8 (1.1-6.5) | 4 (1.5-6.5) | 0.893 |
| average urgency score | 1.3 (0.4-2.2) | 0.9 (0.2-1.7) | 0.8 (0.3-1.8) | 0.9092 |
| days with bloating | 3.8 (0.5-7) | 6 (0.8-6.9) | 5 (0.6-7) | 0.9647 |
| average bloating score | 1.1 (0.1-2.2) | 1.5 (0.2-2) | 0.9 (0.1-1.8) | 0.8297 |

*SLC6A4* (5-HTTPLR)

| **Variable** | **l/l genotype (n=35)** | **l/s genotype (n=43)** | **s/s genotype (n=28)** | ***P* value** |
| --- | --- | --- | --- | --- |
| responder (n=24), % | 34 | 41 | 25 | 0.3719 |
| non-responder (n=80), % | 33 | 33 | 33 |  |
| average stool form, mean (SD) | 4.3 (1.3) | 3.9 (1.2) | 3.7 (1.2) | 0.1075 |
| average stool frequency | 1.7 (1.3-3.1) | 1.8 (1.3-3) | 1.7 (1.4-2.3) | 0.744 |
| days with abdominal pain | 5.5 (2-6.5) | 5 (1.5-6.5) | 4.8 (2.1-7) | 0.916 |
| average abdominal pain score | 0.9 (0.4-1.9) | 0.9 (0.2-1.7) | 1.1 (0.3-1.9) | 0.8309 |
| days with urgency | 5 (1.5-6.5) | 4 (2.5-6.5) | 3.5 (1.5-5.8) | 0.6796 |
| average urgency score, mean (SD) | 1.1 (0.9) | 1.1 (0.9) | 0.9 (0.7) | 0.634 |
| days with bloating | 3.5 (0.5-6.5) | 5.5 (1.5-7) | 5.5 (1.8-6.9) | 0.5369 |
| average bloating score | 0.7 (0.1-1.7) | 1.3 (0.3-2.2) | 1 (0.4-1.8) | 0.514 |

Data are median (IQR) unless stated. Patient samples were genotyped and tested a) for the effect of genotype on stool responder status using the Chi-squared test and b) with clinical symptoms on ondansetron with one-way ANOVA and Kruskal-Wallis tests for parametric and non-parametric data, respectively.

## Supplementary Table 2 - Correlation of *TPH1* mRNA levels with baseline symptoms and biopsy serotonin

| **Biopsy *TPH1* mRNA Comparator** | **r value** | **Correlation significance (*P*)** |
| --- | --- | --- |
| Baseline average stool form | -0.1224 | 0.3474 |
| Baseline average stool frequency | 0.03528 | 0.7872 |
| Baseline days with abdominal pain | -0.02508 | 0.8504 |
| Baseline average abdominal pain | -0.06969 | 0.5967 |
| Baseline days with urgency | 0.008471 | 0.9492 |
| Baseline average urgency | -0.07247 | 0.5822 |
| Biopsy 5-HT | 0.1654 | 0.2274 |
| Biopsy 5-HIAA | 0.1519 | 0.2683 |
| Biopsy 5-HIAA /5-HT | 0.005208 | 0.9708 |

*P* values obtained from Pearson correlation coefficients for parametric data and Spearman correlation for nonparametric data. 5-H: serotonin, 5-HIAA: 5Hydroxyindoleacetic acid, TPH1: Tryptophan hydroxylase 1

## Supplementary Table 3 - Comparison of variables between patients taking <4mg and ≥4mg ondansetron daily

| **Variable** | **<4mg ondansetron** | **≥4mg ondansetron** | ***P* value** |
| --- | --- | --- | --- |
| Age | 39 (12) | 42 (12) | 0.1961 |
| Baseline PHQ-12 | 7.7 (3.5) | 7.3 (3.7) | 0.5644 |
| Baseline PSS | 18 (7) | 18 (9) | 0.5940 |
| Baseline HADS | 15 (7) | 15 (8) | 0.9171 |
| Baseline IBS-SSS | 299 (81) | 297 (90) | 0.9065 |
| Baseline days with abdominal pain, median (IQR) | 5 (3-7) | 6 (4-7) | 0.3098 |
| Baseline average abdominal pain score | 1.2 (0.7) | 1.4 (0.8) | 0.3945 |
| Baseline days with urgency, median (IQR) | 6 (4-7) | 7 (5-7) | 0.1206 |
| Baseline average urgency score | 1.4 (0.6) | 1.7 (0.8) | 0.079 |
| Baseline days with bloating, median (IQR) | 6 (3-7) | 6 (3-7) | 0.5751 |
| Baseline average bloating score, median (IQR) | 1.3 (0.7-1.9) | 1.4 (0.4-2) | 0.8356 |
| Baseline average stool form (BSFS) | 5.1 (0.7) | 5.4 (0.7) | 0.0159 |
| Baseline average stool frequency, median (IQR) | 2.6 (2-4) | 2.9 (2.1-3.9) | 0.7995 |

Data are mean (SD) unless stated. *P* values obtained from unpaired t-tests and Mann-Whitney tests for parametric and non-parametric data, respectively. PHQ-12: Patient Health Questionnaire 12 Somatic Symptoms, PSS: Perceived Stress Scale, HADS: Hospital Anxiety and Depression Scale, IBS-SSS: Irritable Bowel Syndrome Severity Scoring System.

## Supplementary Table 4 - Comparison of variables between patients classed as FDA stool consistency responders and non-responders

| **Variable** | **Stool Consistency responder (n=82)** | **Stool Consistency non-responder (n=25)** | ***P* value** |
| --- | --- | --- | --- |
| Age | 40 (12) | 45 (11) | 0.0539 |
| Gender (women), N (%) | 61 (74) | 16 (64) | 0.446 |
| Hospital Anxiety and Depression Scale | 14.7 (7.0) | 17.8 (8.3) | 0.0774 |
| Patient Health Questionnaire 12 | 7.4 (3.4) | 8.2 (3.9) | 0.3189 |
| Placebo colonic transit, median (IQR) | 18 (9-31) | 10 (6.3-24.8) | 0.0625 |
| Biopsy 5-HT, median (IQR) | 28.6 (18.3-42) | 44.3 (25.5-65.7) | 0.0642 |
| Biopsy 5-HIAA , median (IQR) | 2.1 (1-4.1) | 1.55 (1-4) | 0.6567 |
| Biopsy 5-HIAA /5-HT, median (IQR) | 0.06 (0.03-0.20) | 0.03 (0.02-0.08) | 0.2731 |
| Plasma 5-HIAA , median (IQR) | 16.5 (13.1-19.8) | 16.3 (12.7-18) | 0.7022 |

Data are mean (SD) unless stated. *P* values obtained from unpaired t-tests and Mann-Whitney tests for parametric and non-parametric data, respectively

## Supplementary Table 5 *- TPH1* rs211105 SNP genotypes showing no effect on patient *TPH1* mRNA levels

|  | **TT genotype** | **GT genotype** | **GG genotype** | ***P* value** |
| --- | --- | --- | --- | --- |
| *TPH1* mRNA level | 0.66 (0.33) | 0.70 (0.30) | 0.59 (0.22) | 0.7801 |

Data are mean (SD). TPH1 - Tryptophan hydroxylase 1. *P* value obtained from one way ANOVA

## Supplementary Table 6 - *TPH1* rs4537731 SNP genotypes showing no effect on patient *TPH1* mRNA levels

|  | **TT genotype** | **CT genotype** | **CC genotype** | ***P* value** |
| --- | --- | --- | --- | --- |
| *TPH1* mRNA level | 0.60 (0.21) | 0.71 (0.37) | 0.67 (0.28) | 0.4893 |

Data are mean (SD). TPH1 - Tryptophan hydroxylase 1. *P* value obtained from one way ANOVA

## 5-HT and 5-HIAA analysis methods

Quantification of plasma 5-HIAA and 5HT were carried out on the same high-performance liquid chromatography (HPLC) system. Plasma samples were sonicated in 0.2M perchloric acid for 30 seconds containing 0.1% sodium metabisulfite, and centrifuged at 15 000 *g* for 15 min at 4°C.

Detection and subsequent quantification of 5-HIAA and 5-HT in the supernatant involved the use of reverse-phase, ion-pair (HPLC) coupled with electrochemical detection. Briefly, the method employed a TARGA (75×2.1 mm internal diameter; Higgins Analytical CA). A solvent delivery pump (L-7110, Merck Hitachi, Poole, U.K) was used to circulate mobile phase (0.15 M sodium dihydrogen orthophosphate, 1 mM EDTA, 1.0 mM 1-octane sulphonic acid sodium salt, 14% methanol, adjusted to pH 4.7 with *o*-phosphoric acid, filtered and degassed). Samples were injected onto the column via a Perkin Elmer autosampler series 200 (Bucks, U.K) with a cooling tray set at 4°C. An electrochemical detector (Antec Leyden, Netherlands).The flow rate was 0.15 mL/min and the glassy carbon working electrode potential was set + 0.70 V with reference to a saturated KCl-filled Ag/AgCl reference electrode. The current produced was monitored by ‘System Gold’ software on an IBM PC, with automated data collection (Analogue Interface Module 406).

The detection limit of the high-performance liquid chromatography system with a signal-to-noise ratio of at least 3 was 2 nmol/L for plasma 5-HT and 4 nmol/L for platelet/biopsy specimen 5-HT. The intra-assay coefficient of variation for 10 nmol/L of plasma 5-HT was 9.3% (10 samples). The intra-assay coefficients of variation for 100 nmol/L and 1000 nmol/L for platelet and biopsy specimen 5-HT were 2.2% and .8%, respectively (each based on 10 samples). The intrassay coefficient of variation for 10nmol/l standard sample of 5HIAA was 2.1%. The interassay coefficient of variability of the 10 nmol/l standard sample over the period of analysis was 2.75%. The detection limit of 5-HIAA was 1 nmol/l, defined as the lowest injected amount that produced a signal to noise ratio of 3.
